# The Treatment of Albuminuria

**Published:** 1877-01-20

**Authors:** 


### THE TREATMENT OF ALBUMINURIA.

Dr. T. Lauder Brunton, editor of the *Practitioner*, says, in an article on albuminuria, that its symptoms are those of anæmia, viz., a pale and pasty complexion of the patient, who, on inquiry, tells you that he is weak, that he is short of breath, and suffers from dyspepsia and nervous weakness; that you may observe œdema of the legs, and you find albumen in the urine.

The causation of albuminuria must be distinguished as:

1. Spurious albuminuria due to white of egg.

2. True albuminuria, in which serum albumen appears in the urine, and which is due to venous congestion or disease of the tubules.

The first indication for treatment, then, is, remove the venous obstruction if you can. The second is, lessen the flow of blood to the kidneys by drawing some of it elsewhere.

The venous obstruction depending on pregnancy will cease at the time of parturition, but it may be diminished by the prone position, while that depending on cardiac disease may be lessened by the use of such drugs as digitalis, which causes the heart to contract more forcibly, and by thus diminishing its orifices may render its valves once more competent.

The second indication is fulfilled by warm clothing, warm baths, and diaphoretics, which draw the blood to the skin, and by purgatives, which cause a greater flow of it towards the intestines.

The third indication is to lessen the anæmia which results from the drain of albumen, and of itself produces so many distressing symptoms and injurious effects.

This indication is fulfilled by the administration of iron, which increases the number of blood corpuscles, and at the same time diminishes the loss of albumen through the kidney. I will not at present attempt to explain how it acts, for this is matter of supposition, and others may be prepared with a more probable hypothesis than I can offer.—*Med. and Surg. Rept.*

